# *Schistosoma japonicum* MiRNA-7-5p Inhibits the Growth and Migration of Hepatoma Cells via Cross-Species Regulation of S-Phase Kinase-Associated Protein 2

**DOI:** 10.3389/fonc.2019.00175

**Published:** 2019-03-22

**Authors:** Chao Hu, Shanli Zhu, Jing Wang, Yu Lin, Li Ma, Liufang Zhu, Pengyue Jiang, Zhengli Li, Weiqing Pan

**Affiliations:** ^1^Institute for Infectious Diseases and Vaccine Development, Tongji University School of Medicine, Shanghai, China; ^2^Department of Tropical Diseases, Second Military Medical University, Shanghai, China

**Keywords:** *Schistosoma japonicum*, microRNA, hepatoma cell, SKP2, cross-species regulation

## Abstract

MicroRNAs (miRNAs) play important roles in human diseases, such as cancer. Human miRNA-7-5p is a tumor suppressor miRNA that inhibits tumor growth by regulating multiple oncogenic signal pathways. Recently, studies revealed that plant miRNAs could regulate mammalian gene expression in a cross-kingdom manner. *Schistosoma japonicum* miRNA-7-5p (designated as sja-miR-7-5p) is conserved between the parasites and mammals. Thus, we investigated whether sja-miR-7-5p has similar antitumor activity to its mammalian counterpart. We first showed that sja-miR-7-5p was detected in host hepatocytes during *S. japonicum* infection. The sja-miR-7-5p mimics significantly inhibited the growth, migration, and colony formation of mouse and human hepatoma cell lines *in vitro*, and induced G1/G0 cell cycle arrest. In a xenograft animal model, the tumor volume and weight were significantly reduced in mice inoculated with hepatoma cells transfected with sja-miR-7-5p mimics compared with those transfected with NC miRNAs. Furthermore, the antitumor activity of sja-miR-7-5p was suggested by cross-species downregulation of the S-phase kinase-associated protein 2 gene in the host. Thus, sja-miR-7-5p is translocated into hepatocytes and exerts its anti-cancer activities in mammals, implying that sja-miR-7-5p might strengthen host resistance to hepatocellular carcinoma during schistosome infection.

## Introduction

The primary pathology of schistosomiasis caused by *S. japonicum* is egg-induced granuloma and fibrosis. The female adult worms living in the host mesenteric veins lay numerous eggs, and most of them are trapped in the liver tissues via the portal venous system, causing a granulomatous reaction and fibrosis. The parasite eggs in the granuloma are surrounded by host cells, including immunocytes, hepatic mesenchymal cells, and hepatocytes (1). Our previous studies indicated that *S. japonicum* secretes many microRNAs (miRNAs), including *Schistosoma*-specific and conserved miRNAs (2), and parasite miRNA-containing exosomes (2).

MiRNAs are a class of highly conserved, small non-coding RNAs, with a length of about 20–24 nucleotides (nt) that post-transcriptionally regulate gene expression through complete or incomplete binding to their target mRNAs (3). MiRNAs have extensive effects on not only physiological processes, but also on the progression of many human diseases, such as cancers (4, 5). Aberrant miRNA expression promotes the occurrence and development of various cancers (6–8); however, some miRNAs can exert therapeutic effects on multiple cancers through regulation of tumor-related genes, including those that control tumor cell growth or apoptosis (9, 10). Interestingly, miRNAs derived from plants can regulate the expression of their target genes in mammals in a cross-kingdom manner (11–13). For example, miR-159 derived from plants was detectable in human sera and inhibited breast cancer growth by targeting the human transcription factor 7 (*TCF7*) gene (13). Accumulating evidence indicates that heterogeneous miRNAs can modulate cell functions in mammals. However, it remains unclear how the plant miRNAs can survive the passage through the gastrointestinal tract following ingestion.

Unlike plant miRNAs, which need to pass through the gastrointestinal tract before release into the host serum or entering host cells, schistosomal miRNAs from eggs trapped in liver tissue may be directly transferred to the neighboring host cells. Thus, we hypothesized that parasite miRNAs from the eggs might be translocated into neighboring hepatocytes to exert various biological effects, including some that are beneficial to the host, for example, strengthening the resistance of the host to diseases such as cancer, as do plant-derived miRNAs (13). Human miRNA-7-5p (designated as hsa-miR-7-5p) is a tumor suppressor miRNA that regulates multiple oncogenic signal pathways and reverses drug resistance in certain cancers (14–17). Our previous study identified a *S. japonicum* miRNA-7-5p (designated as sja-miR-7-5p) that is conserved between the parasite and mammals, i.e., there is an identical seed sequence (2–8 nt at the 5′ region) in both parasites and mammalian miRNA-7-5p, despite there being 6 nt differences in the rest of the sequence. Thus, it would be interesting to investigate if sja-miR-7-5 secreted by *S. japonicum* has a similar antitumor activity to hsa-miR-7-5p. In the present study, we demonstrated that sja-miR-7-5p is present in hepatocytes during the *S. japonicum* infection and the sja-miR-7-5p exerts anticancer effects on multiple hepatoma cells (assessed using *in vitro* and *in vivo* models) by targeting the S-phase kinase-associated protein 2(*SKP2*) gene, which is a component of the SCF (Skp1-Cullin 1-F-box) E3 ubiquitin-ligase complex. Previous studies have shown that overexpression of the *SKP2* gene was observed in many cancers, such as in liver cancer (18), prostate cancer (19), lymphoma (20), melanoma (21), and breast cancer (22), which plays an important role in regulating cellular proliferation and cancer progression, mainly by targeting cell cycle regulators in an ubiquitin-dependent manner, followed by 26S proteasome degradation (23). In addition, the *SKP2* overexpression also enhanced tumor cell invasion (24), metastasis (25), and resistance to apoptosis (26), and was associated with tumor aggressiveness (27) and poor prognosis (28).

## Materials and Methods

### Infection of Mice With *S. japonicum* Cercariae

Animal experiments were performed in accordance with the Guide for the Care and Use of Laboratory Animals of the National Institutes of Health, and approved by the Internal Review Board of Tongji University School of Medicine. The animal surgeries were undertaken under sodium pentobarbital anesthesia. Cercariae of *S. japonicum* were provided by National Institute of Parasitic Disease, Chinese Center for Disease Control and Prevention (CDC). 36 six-week-old male C57BL/6J mice (18–20 g, 3 mice per group), purchased from experimental animal center of the Second Military Medical University and housed under specific pathogen-free conditions, were percutaneously infected with 50 or 100 cercariae of *S. japonicum* per mouse (50 for collection of infected hepatocytes and 100 for collection of early stage parasites). For collection of parasites, the hepatic schistosomula were isolated from the portal system and mesenteric veins of infected mice at 7, 14, and 42 days post-infection (dpi). In addition, 42 days male and female adult worms were manually separated under a light microscope. The eggs were isolated with a traditional method, as described by Cai et al. (29). All the freshly isolated parasites were washed three times with PBS (pH 7.4) and were immediately used for extraction of total RNA or frozen at −80°C until being subjected to further analysis.

### Isolation of Primary Mouse Hepatocytes

The primary mouse hepatocytes were isolated by a two-step collagenase perfusion procedure, as described by He et al. (30) with minor modifications. Briefly, after infection, livers of the infected mice collected at various time points of 7, 9, 11, 14, 28, and 42 dpi (*n* = 5) along with the livers of uninfected mice were initially *in situ* digested with 0.03% collagenase type IV and then further digested with 0.08% collagenase type IV at 37°C in a shaking bath for 30 min. The single cell suspensions were harvested by filtration through 400-mesh sieves for removal of the remaining tissue debris and parasite eggs. Next, hepatocytes were isolated by centrifugation of the resulting cell suspensions at 50 × g for 4 min and further purified by centrifugation at 50 × g for 4 min. Purified hepatocytes were resuspend in DMEM containing 20 μg/ml Ribonuclease A (Sigma-Aldrich, USA) at 37°C for 30 min to eliminate any miRNA that might be released by schistosome eggs. After washing with PBS for three times, the cell pellet was immediately used for extraction of total RNA or frozen at −80°C until used.

### Cell Proliferation Assay

Cells (2 × 10^5^) were seeded in a 6-well plate overnight, respectively. Then cells were transfected with sja-miR-7-5p mimics or NC mimics, respectively, four replicates per group. And 24 h later, cells were digested and seeded in a 96-well plate (2 × 10^3^) for 1, 2, 3, and 4 d. At each indicated time, 10 μL Cell Counting Kit-8(CCK-8, Dojindo, Japan) was added to each well and cells were incubated for 1 h at 37 °C, then, using the Microplate reader (Bio-Tek, USA) to measure the abosorbance at 450 nm.

### Cell Cycle Analysis

Cells (1 × 10^5^) were seeded in a 12-well plate overnight, respectively. Then, cells were transfected with sja-miR-7-5p mimics or NC mimics, respectively, three replicates per group. And 48 h later, cells were collected and fixed with ice-cold 75%(v/v) ethanol and stored at 4°C overnight, then, cells were washed and resuspended in 200 μL phosphate-buffered saline (PBS) contained with 0.05 mg/mL RNase A (Beyotime, China) and 25 mg/mL propidium iodide (PI) (Beyotime, China), cell cycle was determined by the FACSverse flow cytometer (BD Biosciences, USA).

### Colony Formation Assay

Cells (2 × 10^5^) were seeded in a 6-well plate overnight, then cells were transfected with sja-miR-7-5p mimics or NC mimics, respectively. And 24 h later, cells were digested and 200 cells in 500 μL complete medium were seeded in 24-well plate, three replicates per group. After incubation for 8 days, then cells were fixed in methanol for 30 min, followed by staining in crystal violet for 15 min. The number of colonies containing > 50 cells was counted under a light microscope.

### Tumor Xenograft Animal Model

Male athymic nude mice were housed and manipulated according to the protocols approved by the Shanghai Medical Experimental Animal Care Commission. Hepa1-6 cells or HepG2 cells were transfected with sja-miR-7-5p mimics or NC mimics, respectively. And 24 h later, for each mouse, 1 × 10^6^ cells in 100 μL PBS after treated with sja-miR-7-5p mimics were injected subcutaneously to the left scapula, while cells after treated with NC mimics were injected subcutaneously to the right scapula, respectively. Tumor volume was measured at 2, 4, 6, and 7 d after injection, At day 7, the mice were sacrificed and tumors were separated to measure their weight and volume. Tumor volume was measured using the formula: 0.5 × L × S^2^, where L is the longest diameter of tumor and S is the shortest diameter of tumor. The content of sja-miR-7-5p mimics transfected into the tumor cells measured by quantitative real-time reverse transcription PCR (qRT-PCR), the protein level of SKP2 was determined by Western blotting. And also the expression of Ki67 in the tumor was measured by immunohistochemistry (IHC) as described under this section.

### Immunohistochemistry

To determine Ki67 expression in xenograft tumor tissues from the athymic nude mice, immunohistochemistry (IHC) was performed as described previously (31), Antibody against Ki67 was used (1:50 dilution).

### Statistical Analysis

All experiments were performed in triplicate and the results were presented as mean ± standard deviation (mean ± SD). All data were analyzed by one-way ANOVA using the software GraphPad Prism 5.0(GraphPad Software, Inc. La Jolla, CA, USA). A value of *P* < 0.05 was considered statistically significant.

## Results

### Presence of sja-miR-7-5p in Infected Hepatocytes

**Figure 1:**
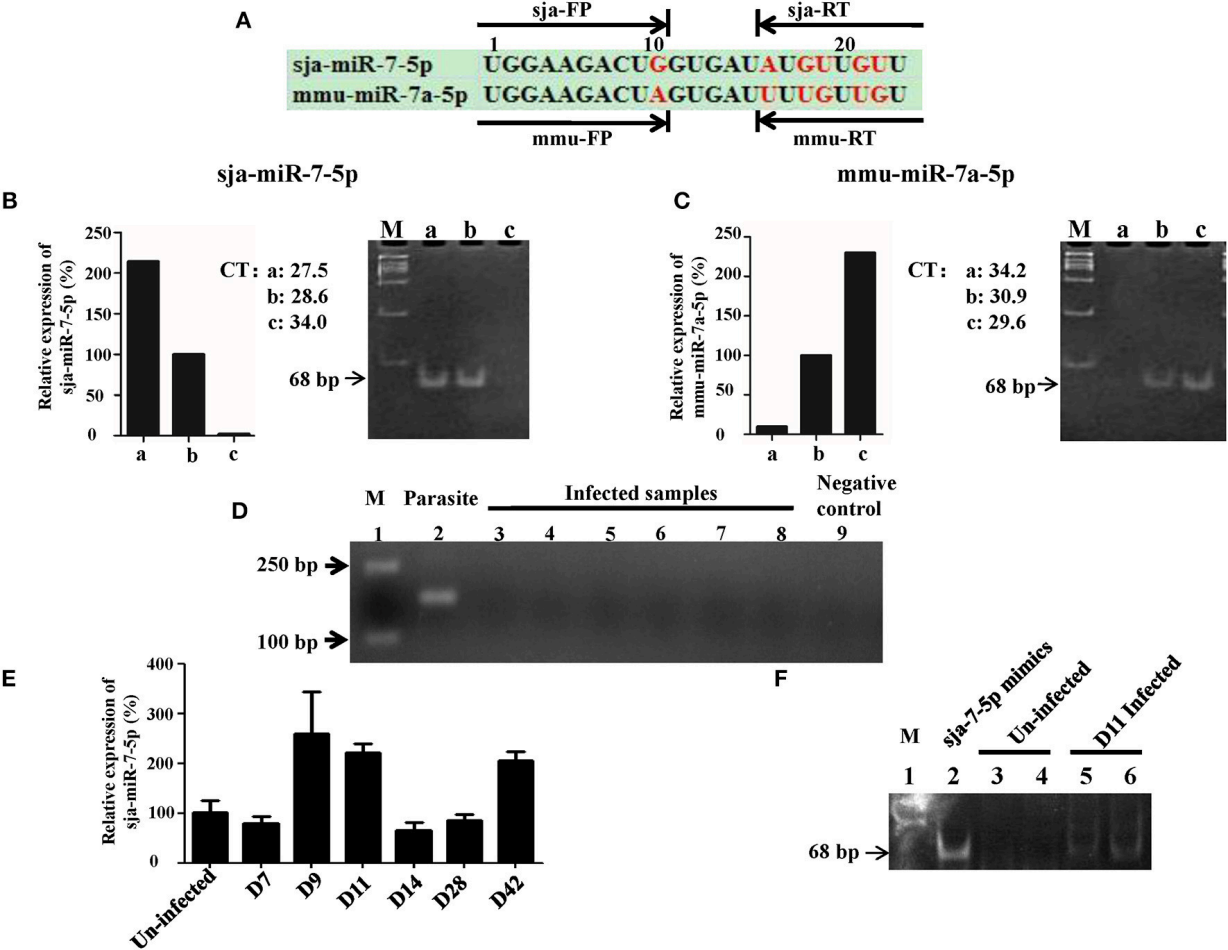
Detection of sja-miR-7-5p in infected hepatocytes**. (A)** A schematic diagram represents two sets of primers of reverse transcription stem-loop primer (RT) and forward primer (FP) for sja-miR-7-5p or mmu-miR-7a-5p, respectively. **(B,C)** Preparation of RNA samples: a. 200 ng *Schistosoma japonicum* egg RNA; b. mixture of equal amount of *Schistosoma japonicum* egg RNA (100 ng) and Hepa1-6 cell RNA (100 ng); c. 200 ng Hepa1-6 cell RNA. The three RNA templates were transcribed into cDNA using the corresponding reverse transcription stem-loop primer, respectively, which were used for qRT-PCR by the corresponding forward primer and common reverse primer, respectively. The PCR products were separated by polyacrylamide gel electrophoresis (PAGE). As shown in B and C, the two sets of primers can effectively distinguished the sja-miR-7-5p and mmu-miR-7a-5p, e.g., the set of sja-miR-7-5p RT and forward primer FP amplified the sja-miR-7-5p [**(B)**, lane a and b] but not mmu-miR-7a-5p (lane c), while the set of mmu-miR-7a-5p RT and forward primer FP generated the mmu-miR-7a-5p but not the sja-miR-7-5p **(C)**. **(D)** Analysis of the RNA samples to ensure no contamination with parasite RNA: the RNA samples used for the above analysis were detected as described in Method by PCR for presence of the NADH gene of *S. japonicum*. Lane 1: marker. Lane 2: parasite positive control: RNA samples of *S. japonicum* eggs as described above. Lane 3–8: six samples of infected hepatocytes with RNase pre-incubation. Lane 9: negative control without the template. **(E)** qRT-PCR analysis of sja-miR-7-5p in the infected hepatocytes at various days after infection; **(F)** 12% PAGE analysis showing sja-miR-7-5p PCR product (68 bp) from the infected hepatocytes: Lane 1: marker; Lane 2: sja-miR-7-5p mimics positive control; Lanes 3 and 4: two uninfected hepatocyte samples with pre-incubation with RNase; Lanes 5 and 6: two infected hepatocyte samples at day 11 post-infection with the pre-incubation. Data are presented as the mean ± SD, *n* = 3.

We first investigated whether sja-miR-7-5p was present in the host liver cells during schistosome infection. For this purpose, we designed a set of two sets primers that could distinguish the sja-miR-7-5p from corresponding miRNA derived from mouse (mmu-miR-7-5p) and human (hsa-miR-7-5p). The sja-miR-7-5p has an identical seed sequence (2–8 nt at the 5' region), but there are 6 nt differences in the rest of the sequence among the species (Figure 1A, and Figure S1), which allowed us to design sets of specific primers for the mmu-miR-7a-5p (mmu-FP/RT) and the sja-miR-7-5(sja- FP/RT). We first tested specificity of the primers using the RNA samples derived from *S. japonicum* eggs (a), mouse Hepa1-6 cell line (c), and mixture of equal amount of a and c (b). As shown in Figures 1B,C, the sja-FP/RT pair primers successfully generated the sja-miR-7a-5p from the samples of a and b, but not c (Figure 1B), while the mmu-FP/RT pair primers generated the mmu-miR-7a-5p from the sample b and c, but not a (Figure 1C). These data indicated that the two sets of primers can effectively distinguish the sja-miR-7-5p and mmu-miR-7a-5p, and no cross reaction between the mmu-FP/RT and sja-FP/RT primers. All the primers are listed in Table S1.

We next used the sja-FP/RT primers for detection of presence of sja-miR-7-5p in the liver cells of infected mice with *S. japonicum*. We prepared RNA samples from the infected liver cells, and carefully analyzed the samples to ensure no contamination with parasite RNA (Figure 1D). We showed that sja-miR-7-5p was detected by using qRT-PCR in the hepatocytes from infected mice at the early stage (i.e., days 9 and 11 post infection) and the late stage of infection (day 42) (Figure 1E). The presence of this parasite miRNA was further verified by PCR (Figure 1F) and cloning and sequencing of the PCR product showed identical sequence of sja-miR-7-5p (Figure S2A). In addition, we showed that sja-miR-7-5p was expressed at all these stages, and higher expression of sja-miR-7-5p was detected in adult males compared with that in adult females (Figure S2B). These findings indicated that this sja-miR-7-5p is present in the host liver cells during schistosome infection.

### Inhibition of Proliferation and Migration of Hepatoma Cells by Sja-miR-7-5p

**Figure 2:**
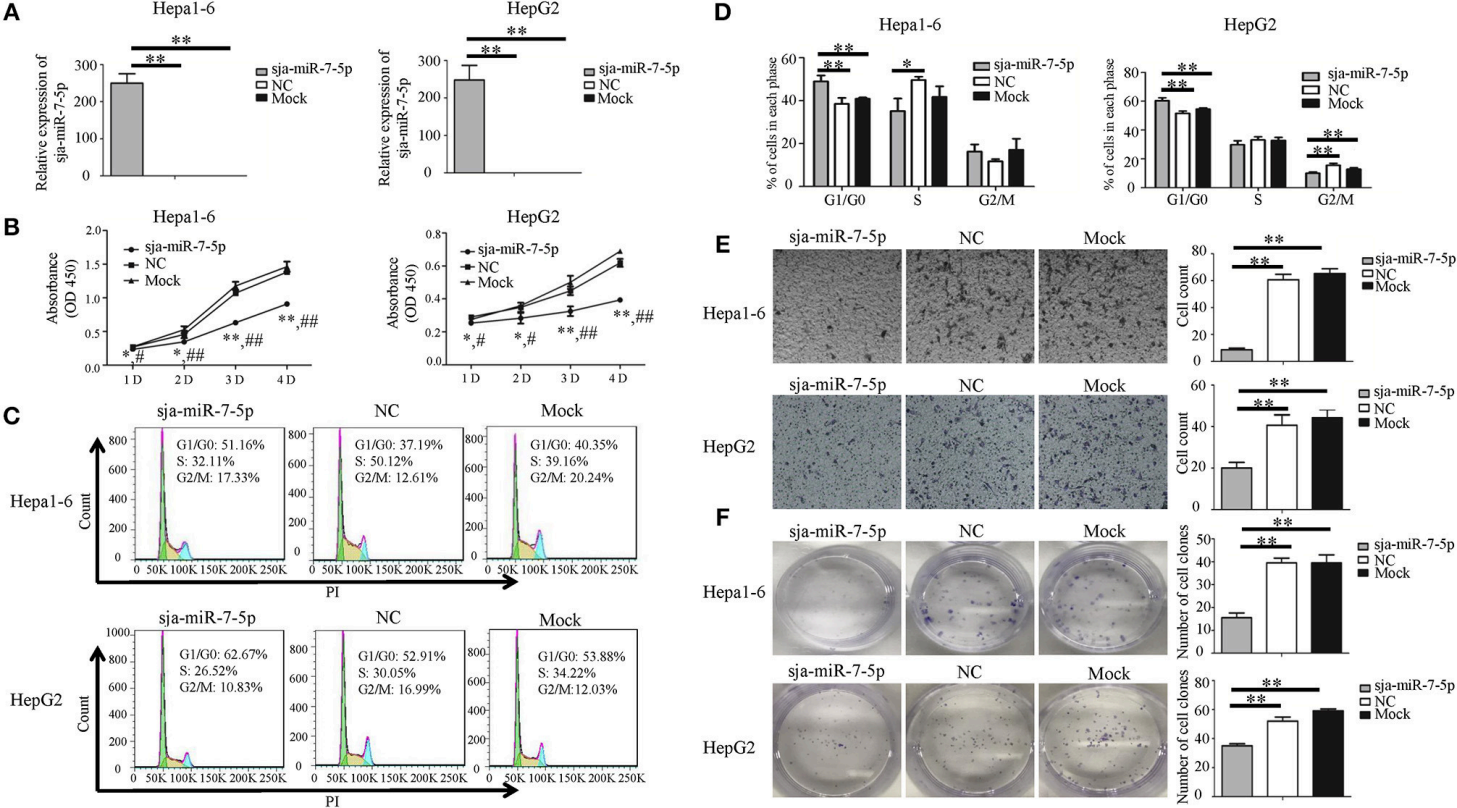
Sja-miR-7-5p inhibits cell proliferation and migration of Hepa1-6 and HepG2 cells *in vitro***. (A–F)** Hepa1-6 and HepG2 cells were transfected with sja-miR-7-5p mimics and NC (negative control) mimics, respectively, and 48 h later [except for the cell counting kit-8(CCK-8) assay, which was 24 h later], the expression of sja-miR-7-5p was determined using qRT-PCR (A). Cell proliferation was evaluated by CCK-8 assay at 1, 2, 3, and 4 days **(B)**, data are presented as the mean ± SD, *n* = 3, ^*^p < 0.05 or ^**^p < 0.01 indicates the comparison between the two groups of sja-miR-7-5p and NC; #*p* < 0.05 or ##*p* < 0.01 indicates the comparison between the groups of sja-miR-7-5p and Mock. Cell cycle was determined by flow cytometry analysis **(C,D)**. Cell migration was evaluated using Transwell inserts without matrigel coating **(E)**. The ability to form cell clones was determined using a colony formation assay **(F)**. Data are presented as the mean ± SD, *n* = 3, ^*^p < 0.05, ^**^p < 0.01.

To investigated the effects of sja-miR-7-5p on the growth of hepatoma cells *in vitro*, both mouse and human hepatoma cells (e.g., Hepa1-6 cells and HepG2 cells) were transfected with the sja-miR-7-5p mimics, NC (a negative control mimics that has no target gene in mice and human) and Mock (transfection reagents only). As shown in Figure 2A, the sja-miR-7-5p mimics were effectively transfected into both cell lines. The schistosomal miRNA significantly suppressed the proliferation of both cell lines, as measured by the CCK-8 assay (Figure 2B), and substantially arrested the cell cycle at G1/G0 phase, as detected by flow cytometry (Figures 2C,D). We also showed that transfection of the sja-miR-7-5p mimics significantly suppressed cell migration, as assessed using the Transwell inserts without matrigel coating (Figure 2E) and by the wound-healing assay (Figures S3B,C) compared with the NC or Mock control cells. Colony formation assays showed that sja-miR-7-5p inhibited colony formation of hepatoma cells to a greater extent that those in the NC group or Mock group (Figure 2F). In addition, the Hepa1-6 cells transfected with sja-miR-7-5p mimics grew bigger and rounder compared with those in the NC or Mock control cells (Figure S3A). These data indicated that sja-miR-7-5p inhibited growth, migration, and colony formation of both mouse and human hepatoma cells and arrested their cell cycle at G1/G0 phase *in vitro*, indicating that the schistosomal miRNA is also a tumor suppressor.

### Sja-miR-7-5p-Mediated Inhibition of Hepatoma Cell Growth *in vivo*

**Figure 3:**
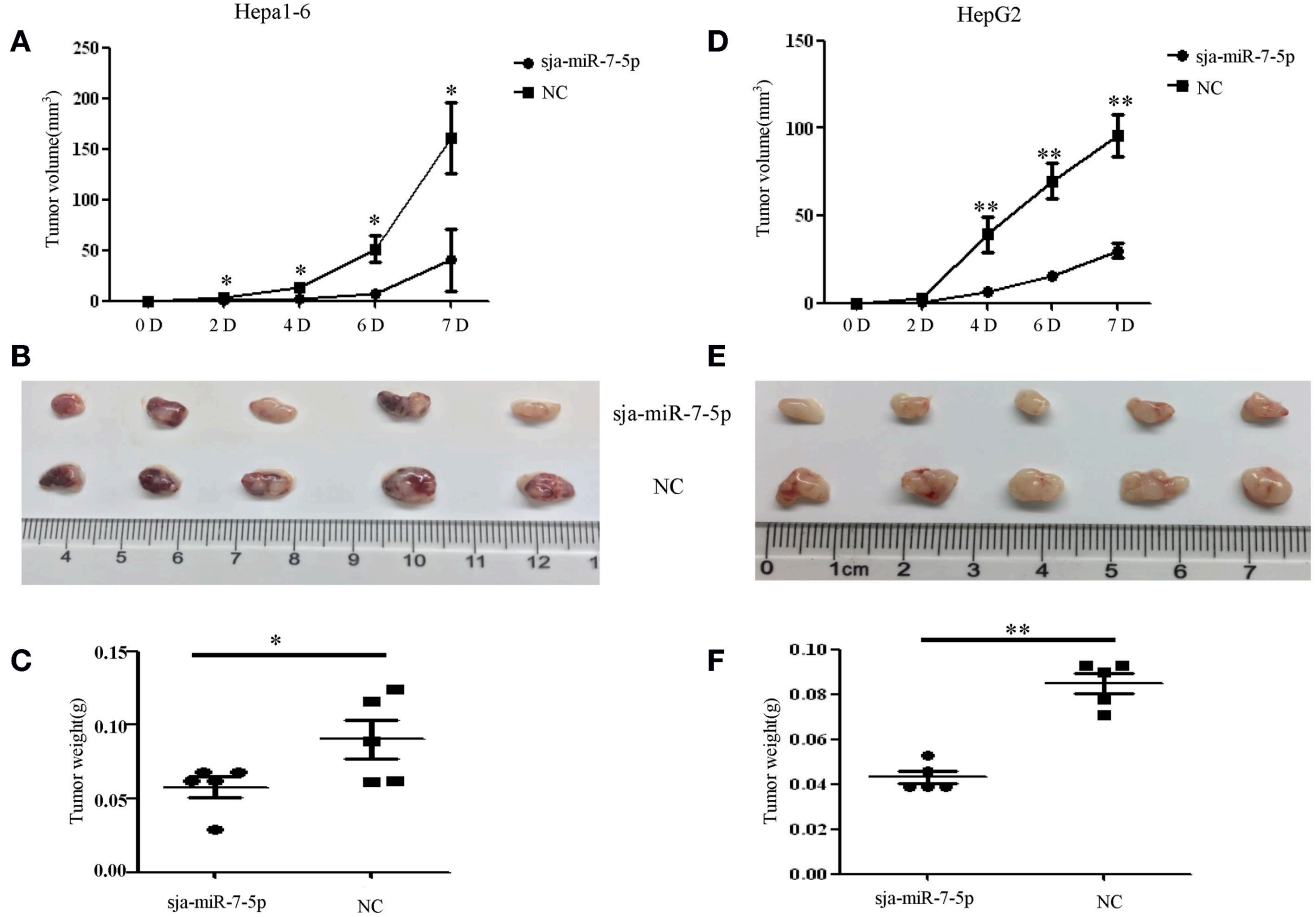
Sja-miR-7-5p inhibits hepatoma cell growth *in vivo***. (A–F)** Hepa1-6 and HepG2 cells were transfected with sja-miR-7-5p mimics or NC mimics, respectively, and then the sja-miR-7-5p-treated cells (1 × 10^6^) were injected subcutaneously to the left scapula of athymic nude mice, and the NC-treated cells were injected subcutaneously into the right scapula (*n* = 5), respectively. Tumor volumes were measured at days 2, 4, 6, and 7 after injection. At day 7, the mice were sacrificed and tumors were separated to measure their weight and volume, **(A–C)** for Hepa1-6 cells, **(D–F)** for HepG2 cells. Data are presented as the mean ± SD, *n* = 5, ^*^p < 0.05, ^**^p < 0.01.

To further investigate whether sja-miR-7-5p inhibits growth of liver cancer cells *in vivo*, both Hepa1-6 and HepG2 cells were transfected with sja-miR-7-5p mimics or NC mimics, and then injected subcutaneously to the left and right scapula of athymic nude mice to generate subcutaneous tumors. The tumor volume was measured at days 2, 4, 6, and 7 after injection. At day 7, mice were sacrificed and tumors were excised to measure their weight and volume. The results showed that both the tumor volume and weight were significantly reduced in the mice inoculated with Hepa1-6 cells transfected with sja-miR-7-5p mimics compared with those in mice receiving cells transfected with NC miRNAs (Figures 3A–C). Similar results were obtained with the human cell line of HepG2 (Figures 3D–F). These data indicated that sja-miR-7-5p suppressed tumor growth *in vivo*.

### *SKP2* Is a Direct Target of Sja-miR-7-5p

To determine the molecular mechanisms by which sja-miR-7-5p inhibits hepatoma cell growth, we used the online software miRDB (32) (http://www.mirdb.org/miRDB/index.html), MR-microT (33) (http://diana.imis.athena-innovation.gr/DianaTools/index.php?r=mrmicrot/index) and RNAhybrid (34) (http://bibiserv.techfak.uni-bielefeld.de/rnahybrid?id=rnahybrid_view_submission).

To search for potential targets of sja-miR-7-5p. We identified the gene encoding S-phase kinase-associated protein 2(*SKP2*) as a potential target for sja-miR-7-5p, because a binding site was located at the 3' UTR of the both murine and human *SKP2* gene that perfectly matched the seed sequence of sja-miR-7-5p. In addition, the *SKP2* gene in human has been characterized as an oncogene during tumorigenesis (21, 35–38).

**Figure 4:**
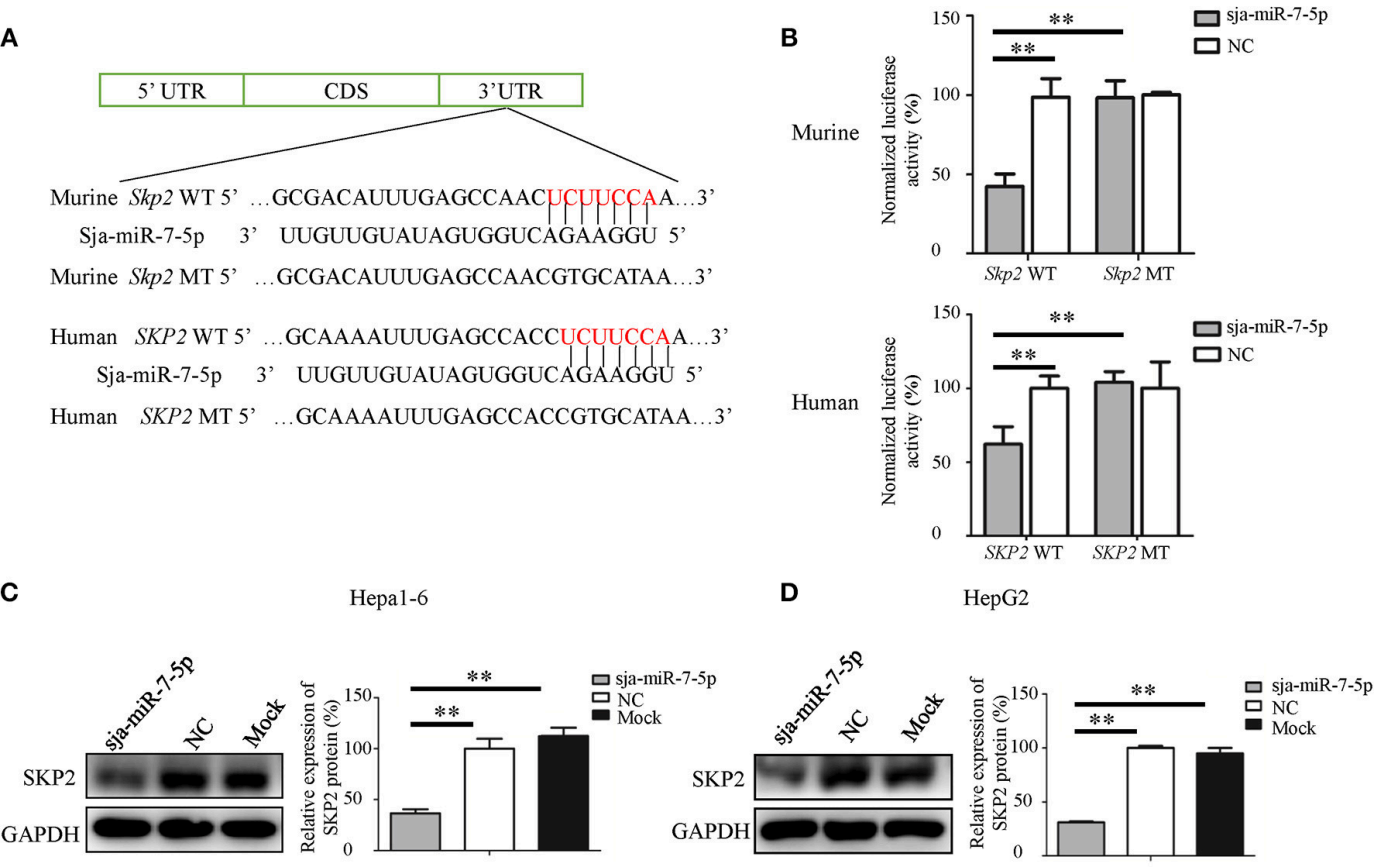
*SKP2* (encoding S-phase kinase-associated protein 2) is a direct target of sja-miR-7-5p. **(A)** A schematic diagram representing the wild-type or mutant 3' untranslated region (UTR) sites of murine *Skp2* and human *SKP2* genes targeted by sja-miR-7-5p. **(B)** A dual-luciferase reporter assay was used to measure the activity of the reporter gene, and the firefly luciferase activity was normalized to renilla luciferase activity. **(C,D)** The protein levels of murine SKP2 **(C)** and human SKP2 **(D)** were measured using western blotting after transfection with sja-miR-7-5p mimics or NC mimics, respectively. Data are presented as the mean ± SD, *n* = 3, ^**^p < 0.01.

To investigate the relationship between sja-miR-7-5p and *SKP2* gene in both human and mouse, first, we constructed two plasmids that contain the luciferase reporter gene: One was the pmirGLO-*SKP2*-WT construct in which the firefly luciferase gene is fused to the 3' UTR of *SKP2* gene; the other was the pmirGLO-*SKP2*-MT in which the seven nucleotides in the miRNA binding site were mutated (Figure 4A). The constructs were simultaneously transfected with sja-miR-7-5p mimics or NC mimics into both Hepa1-6 cells and HepG2 cells. As shown in Figure 4B, the luciferase activity was significantly decreased in the cells transfected with the pmirGLO-*SKP2*-WT but not with the pmirGLO-*SKP2*-MT, indicating that sja-miR-7-5p mimics could directly bind to the site in the 3′ UTR of the *SKP2* gene, while the mutations in the seed sequence abrogated the inhibitory effect.

We then detected the level of the SKP2 protein in both Hepa1-6 or HepG2 cells transfected with sja-miR-7-5p mimics using Western blotting. We found that sja-miR-7-5p downregulated the levels of SKP2 in both Hepa1-6 cells and HepG2 cells compared with that in cells transfected with NC or Mock controls (Figures 4C,D).

### Sja-miR-7-5p-Mediated Suppression of the Hepatoma Cell Growth Through Downregulation of SKP2 Expression

**Figure 5:**
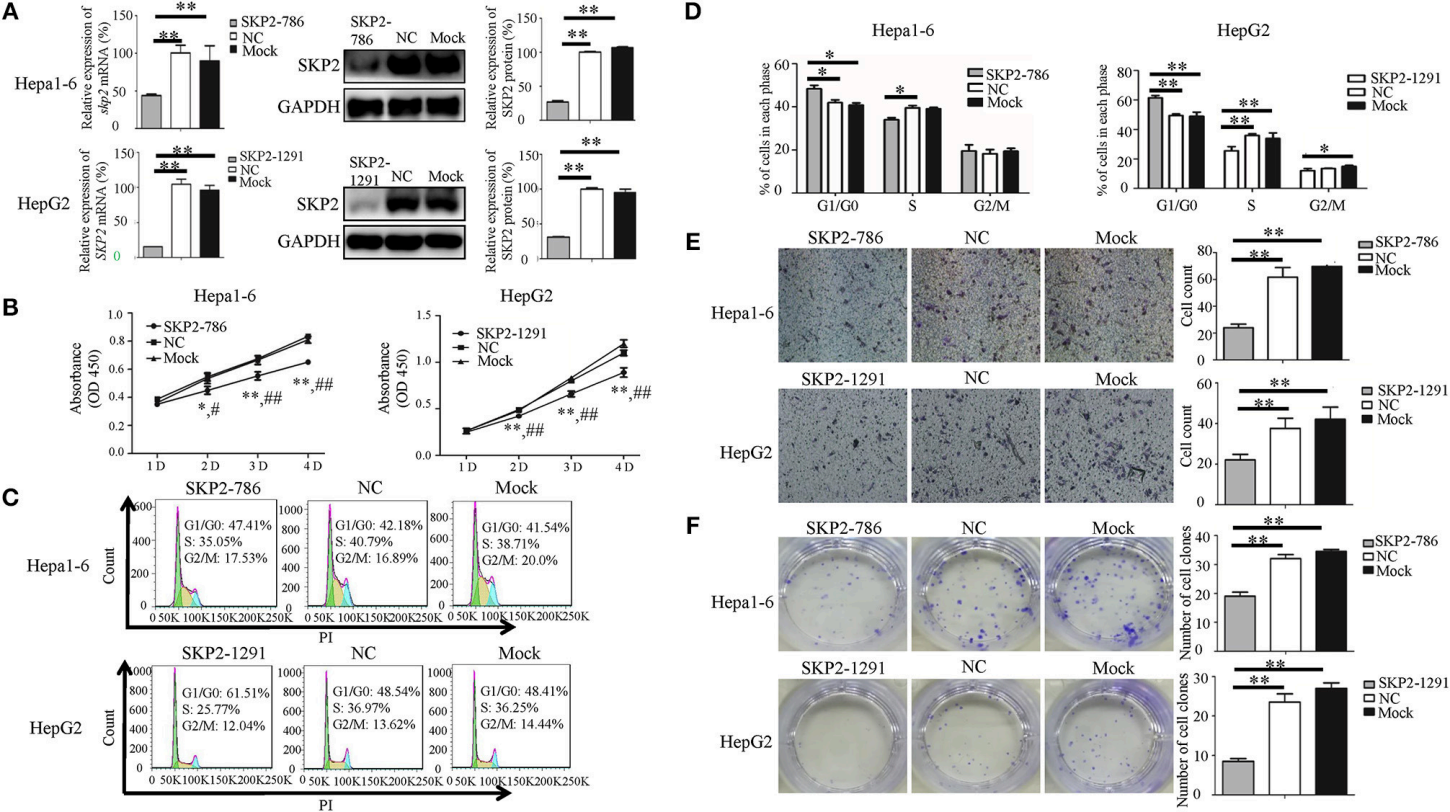
Knockdown of *SKP2* inhibits cell proliferation and migration of Hepa1-6 and HepG2 cells *in vitro*. **(A–F)** Hepa1-6 and HepG2 cells were transfected with *SKP2* siRNA and negative control (NC) siRNA, respectively, and 48 h later (except for the cell counting kit-8(CCK-8) assay, which was 24 h later), the expression of *SKP2* was determined using qRT-PCR and western blotting **(A)**. Cell proliferation was evaluated using the CCK-8 assay at 1, 2, 3, and 4 d **(B)**, data are presented as the mean ± SD, *n* = 3, ^*^p < 0.05 or ^**^p < 0.01 indicates the comparison between the two groups of sja-miR-7-5p and NC; #*p* < 0.05 or ##*p* < 0.01 indicates the comparison between the two groups of sja-miR-7-5p and Mock. Cell cycle was determined using flow cytometry analysis **(C,D)**. Cell migration was evaluated by using Transwell inserts without matrigel coating **(E)**. The ability to form cell clones was determined using a colony formation assay **(F)**. Data are presented as the mean ± SD, *n* = 3, ^*^p < 0.05, ^**^p < 0.01.

To investigate whether sja-miR-7-5p inhibits the growth of hepatoma cells through inhibition of SKP2 expression, both Hepa1-6 cells and HepG2 cells were transfected with the *SKP2* small interfering RNAs (siRNAs). We showed that both murine *Skp2* siRNA (SKP2-786) and human *SKP2* siRNA (SKP2-1291) significantly reduced the SKP2 expression in Hepa1-6 cells and HepG2 cells, respectively, at both transcriptional and translational levels detected by qRT-PCR and Western blotting (Figure 5A). Importantly, similar to the observations in the sja-miR-7-5p mimics-treated cells, the transfected Hepa1-6 cells and HepG2 cells with the siRNA showed cell cycle arrest at the G0/G1 phase (Figures 5C,D), and inhibition of cell proliferation (Figure 5B), cell migration (Figure 5E), and colony formation (Figure 5F), whereas these inhibitory effects were not observed in the cells treated with the NC siRNA. The phenotypes of the cells treated with *SKP2* siRNA were similar to those of sja-miR-7-5p mimics-treated cells, which suggested that the inhibitory effects of the schistosome miRNA on hepatoma cells function by downregulating SKP2 expression.

**Figure 6:**
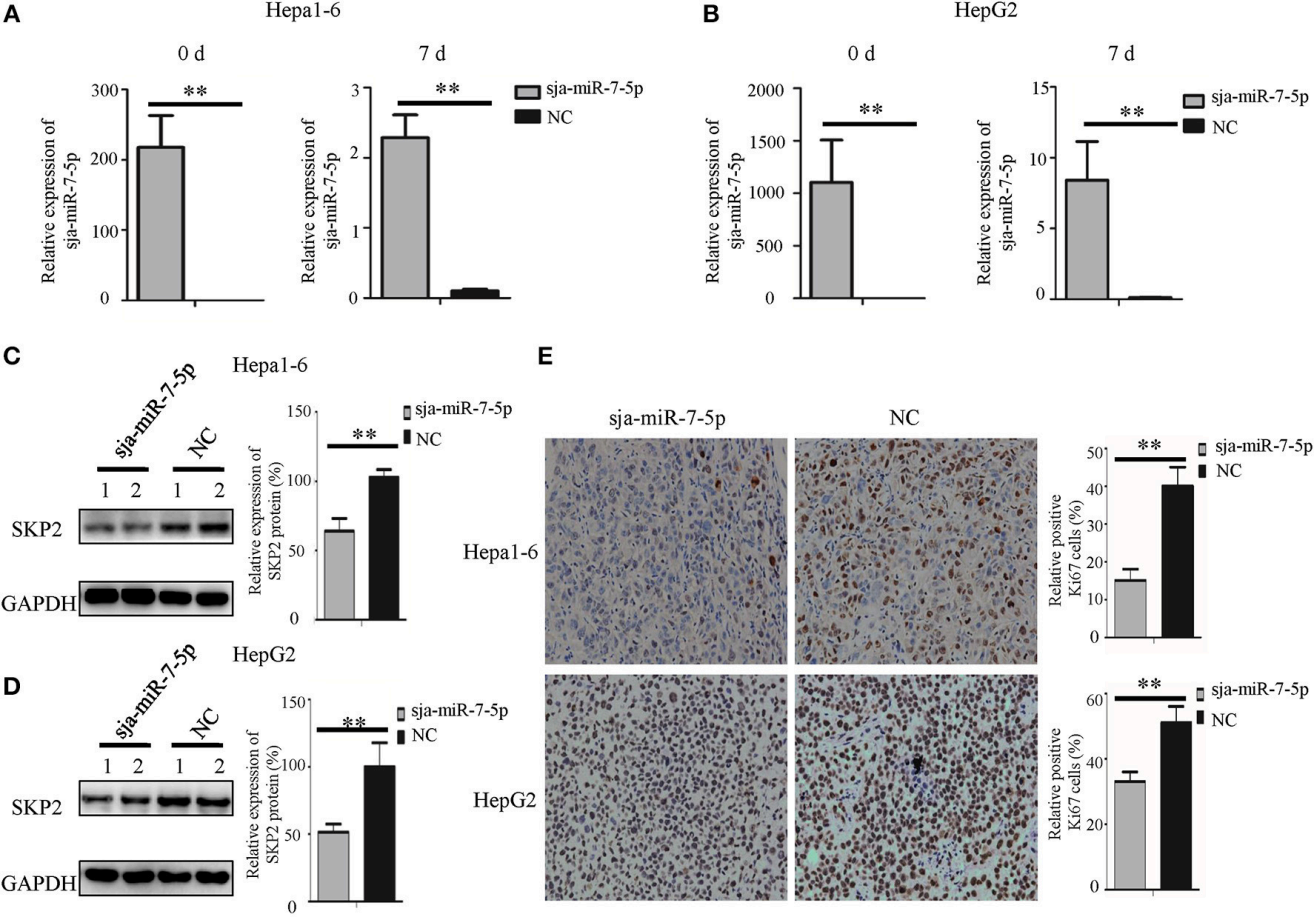
Sja-miR-7-5p inhibits the expression of SKP2 and Ki67 within hepatoma cell tumors. **(A,B)**The content of sja-miR-7-5p mimics after transfection into tumor cells was measured using qRT-PCR before inoculation (0 d) and after sacrifice (7 d), with U6 as the internal control, **(A)** for Hepa1-6 cells, **(B)** for HepG2 cells. Data are presented as the mean ± SD, *n* = 5, ^**^p < 0.01. **(C,D)** The protein level of SKP2 was determined by Western blotting, with glyceraldehyde-3-phosphate (GAPDH) as the internal control, **(C)** for Hepa1-6 cells, **(D)** for HepG2 cells. **(E)** The level of Ki67 in tumors was determined using immunohistochemistry.

We also detected the expression of *SKP2* gene in the subcutaneous tumors generated by Hepa1-6 or HepG2 cells transfected with sja-miR-7-5p or NC mimics, respectively. As shown in Figures 6A,B, the transfected sja-miR-7-5p was detectable in the tumors on day 7 after injection. We then detected the SKP2 protein level using Western blotting, which showed that the level of SKP2 was significantly decreased in the tumors of both Hepa1-6 and HepG2 cells receiving sja-miR-7-5p compared with that in tumors from cells transfected with the NC control (Figures 6C,D). Meanwhile, we evaluated the proliferation of the tumor cells using immunohistochemistry (IHC) for Ki67, which showed that the protein level of Ki67 was also significantly decreased in tumor cells transfected with sja-miR-7-5p compared with that in cells transfected with the NC control (Figure 6E). These data further suggested that sja-miR-7-5p inhibited proliferation of both Hepa1-6 cells and HepG2 cells via downregulation of SKP2 expression.

**Figure 7:**
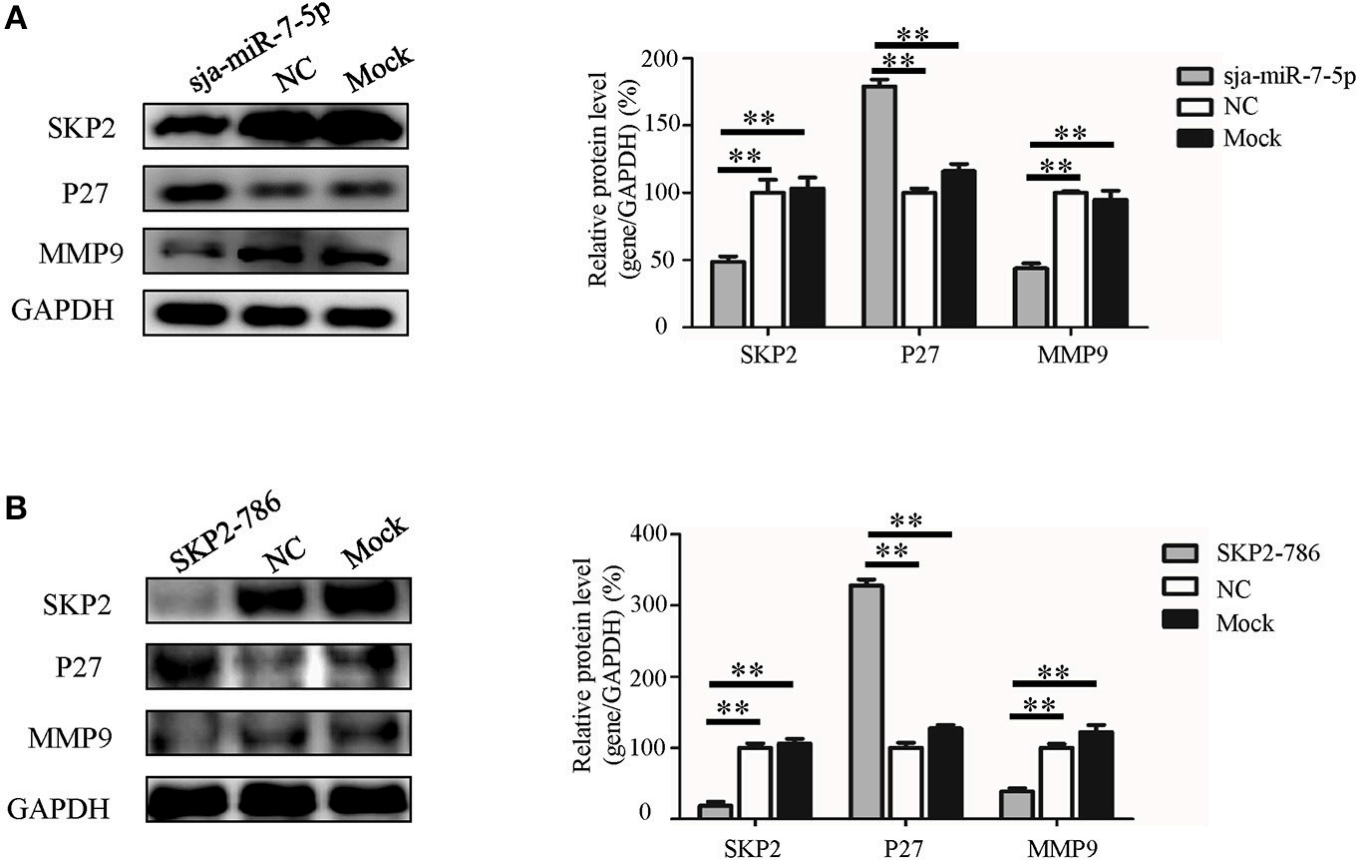
The molecular mechanism whereby sja-miR-7-5p exerts its antitumor activity in hepatoma cells. **(A,B)** In Hepa1-6 cells, the protein levels of SKP2, P27, and MMP9 were measured by Western blotting after transfection with sja-miR-7-5p mimics **(A)** or *Skp2* siRNA **(B)** and their corresponding negative controls, with glyceraldehyde-3-phosphate (GAPDH) as the internal control. Data are presented as the mean ± SD, *n* = 3, ^**^p < 0.01.

To further explore the molecular mechanism by which sja-miR-7-5p exerts its antitumor activities, we detected the expression of two downstream nodes of SKP2, e.g., P27 [also known as cyclin dependent kinase inhibitor 1B (CDKN1B)] and matrix metalloproteinase 9(MMP9), using Western blotting. We found that sja-miR-7-5p downregulated the level of SKP2, which led to significantly increased levels of P27 and reduced levels of MMP9 in the cells receiving sja-miR-7-5p mimics compared with those in the cells receiving the NC mimics (Figure 7A). In addition, transfection of the hepatoma cells with the murine *Skp*2 siRNA generated a similar outcome to that in cells transfected with sja-miR-7-5p mimics (Figure 7B). These data suggested that sja-miR-7-5p exerts its antitumor activity by targeting *SKP2* to elevate P27 levels, which led to suppression of tumor cell growth, and reducing MMP9 levels, resulting in inhibition of cell migration.

## Discussion

Hsa-miR-7-5p is well-characterized as a tumor suppressor miRNA that suppresses survival, proliferation, invasion, and migration of multiple cancer cells, as well as increasing the sensitivity of resistant tumor cells to therapeutics. The molecular mechanism underpinning its anticancer activities involves regulation of multiple signaling related genes such as *PI3K/Akt, FAK, KLF4*, and *REG*γ (10, 39–41). This miRNA has therapeutic potential for human cancers (42). Our previous studies identified a conserved miR-7-5p from *S. japonicum*, sja-miR-7-5p, that has an identical seed sequence to those of hsa-miR-7-5p and mouse mmu-miR-7-5p, although there are 6 nt differences in the rest of the sequence of the miRNA among species. In this study, we have demonstrated that the schistosome miRNA, sja-miR-7-5p, is present in host hepatocytes during schistosome infection, and the *in vitro* transfection of sja-miR-7-5p mimics into hepatoma cells led to cell cycle arrest and inhibition of cell proliferation, colony formation, and cell migration. Furthermore, we showed that sja-miR-7-5p suppressed the growth of both human and mouse hepatoma cells in a xenograft animal model. Analysis of the molecular mechanisms revealed that sja-miR-7-5p exerts its activities by targeting the *SKP2* gene, which is involved in regulation of cell viability and migration. Thus, the present data indicated that the schistosome sja-miR-7-5p is also a tumor suppressor miRNA that may have therapeutic potential for human cancers. In addition, both the presence of this miRNA in host hepatocytes and its antitumor effects on human hepatoma cells suggest that schistosome non-small RNA-mediated anticarcinogenic effects might exist in the host liver during schistosome infection.

Infection with several parasites, such as *Opisthorchis viverrini* and *Clonorchis sinensis*, has been reported to be associated with cancer (43, 44). Schistosomiasis is a neglected tropical parasitic disease, affecting approximately 210 million people worldwide. Infection with *Schistosoma haematobium* is associated with bladder cancer (43, 44). However, for infection with *S. japonicum*, the association with hepatocellular carcinoma (HCC) is less evident, although a potential association with colorectal cancer was reported (45). The large retrospective epidemiological surveys conducted in highly endemic areas for schistosomiasis in China showed no correlation between HCC and *S. japonicum* infection (46). Although several other epidemiological and case–control studies proposed a potential association between HCC and *S. japonicum* infection, the evidences for the association remain a matter of debate because the schistosomiasis patients are highly associated with HBV and HCV infections, which are hepatic carcinogens (47). However, accumulating evidence indicates that chronic inflammation plays an important role in carcinogenesis (48). For *S. japonicum* infection, the liver-trapped eggs induce severe hepatic chronic inflammation and fibrosis that could be risk factors for HCC (49). These factors derived from *S. japonicum* infection should contribute to HCC, but this does not seem to happen in *S. japonicum* schistosomiasis. Therefore, we speculated that the *S. japonicum* eggs trapped in the liver might play a dual role in the HCC occurrence and development, i.e., carcinogenic and anticancer activities, similar to those reported for the protozoan *Trypanosoma cruzi*, which has carcinogenic and anticancer activities during infection (50). This study demonstrated that a non-coding small RNA secreted by *S. japonicum*, sja-miR-7-5p, perhaps together with other miRNAs derived from the parasite, could be translocated into liver cells during parasitic infection, and exerts anticancer activity, implying that the *S. japonicum*-producing non-coding small RNAs may, in part, contribute to the anticancer activities in the infected host.

As described above, mammalian miR-7-5p exerts its anticancer activities through regulation of multiple target genes such as *PI3K/Akt, FAK* and *KLF4*. To identify the target gene of the parasite sja-miR-7-5p, we first used three online software to search for its potential target genes. We found 5 target gene candidates (*Skp2, Psme3, Pik3cd, Klf4, and Hoxb5*) that were consistently predicted by the three software and involved in tumor-related signaling pathway. Three of them (i.e., *Pik3cd, Klf4, Hoxb5*) were excluded through analysis of their expression in hepatoma cells transfected with the sja-miR-7-5p mimics. Although both *Skp2* and *Psme3* genes were validated as target gene by luciferase reporter assay, our experimental data with *Skp2* and *Psme3* siRNA showed that only the hepatoma cell transfected with the *Skp2* siRNA produced similar phenotype to that of sja-miR-7-5p mimics-treated cells. Thus, *Skp2* gene has been identified as the target gene of sja-miR-7-5p.

SKP2, also known as P45, FBL1, FLB1, and FBXL1, is a component of the SCF (Skp1-Cullin 1-F-box) E3 ubiquitin-ligase complex. Many studies have reported that SKP2 is overexpressed in various cancers of different organs, including the liver (18), colon (51), breast (52), prostate (53), and stomach (54). *SKP2* is characterized as an oncogene, and is involved in modulation of the cell cycle, cell growth, and survival by regulation of its downstream node molecules, such as P27, P16, P21, P57, E2F-1, and c-MYC in an ubiquitin-dependent manner, followed by 26S proteasome degradation (23). Previous studies showed that loss of *SKP2* reduced the migration and invasion abilities of oral squamous cell carcinoma cells by downregulating the expression of MMP2 and MMP9 (55). The best-known substrate of SKP2 is the cyclin dependent kinase (CDK) inhibitor, P27. Overexpression of SKP2 leads to reduction of P27, which is strongly associated with aggressive tumor behavior and poor clinical outcome (19, 36, 56), while knockdown of *SKP2* resulted in the accumulation of P27, causing cell cycle arrest at G1/G0 phase (57). However, the relationship between miR-7-5p and *SKP2* has not yet been reported in HCC. In the present study, we found that in liver cancer cells, including Hepa1-6 cells and HepG2 cells, sja-miR-7-5p inhibited the growth and migration of both mouse and human hepatoma cells by targeting *SKP2* to elevate the expression of P27 and decrease the expression of MMP9. These data were consistent with the results of experiments using the *SKP2* siRNA, and with the outcome of a study in which miRNA-7-5p could suppress cell proliferation of CHO cells partly by targeting *skp2* (58). Therefore, our data demonstrated that sja-miR-7-5p suppresses hepatoma cell growth and migration by downregulating *SKP2*.

The present study demonstrated that sja-miR-7-5p is present in infected hepatocytes, selectively affects the growth and migration of human and mouse tumor cells by targeting the *SKP2* gene, implying that sja-miR-7-5p might strengthen resistance of host to cancer during schistosome infection.

## Data Availability

All datasets generated for this study are included in the manuscript and/or the Supplementary Files.

## Author Contributions

CH and WP conceived and designed the study. CH, SZ, JW, YL, LM, LZ, PJ, and ZL performed the experiments. CH, SZ, and WP analyzed the data. CH and WP wrote the manuscript. All authors read and approved the final manuscript.

### Conflict of Interest Statement

The authors declare that the research was conducted in the absence of any commercial or financial relationships that could be construed as a potential conflict of interest.

## Abbreviations

*S. japonicum*: *Schistosoma japonicum*
HCC: hepatocellular cancer
SKP2: S-phase kinase associated protein 2
P27(also known as CDKN1B): cyclin dependent kinase inhibitor 1B
MMP9: matrix metallopeptidase 9
miRNA: microRNA
siRNA: small interfering RNA
NC: negative control
Mock: mock control.
