# Supplementary material for: Schistosoma japonicum MiRNA-7-5p Inhibits the Growth and Migration of Hepatoma Cells via Cross-Species Regulation of S-Phase Kinase-Associated Protein 2
Source: Front Oncol. 2019 Mar 22;9:175. doi: 10.3389/fonc.2019.00175 (PMC6443022; doi:10.3389/fonc.2019.00175)
Supplement: Supplementary file 1 [file Data_Sheet_1.docx]

**Title:**

***Schistosoma japonicum* MiRNA-7-5p Inhibits the Growth and Migration of Hepatoma Cells via Cross-species Regulation of S-phase Kinase-associated Protein 2**

**Authors:**

Chao Hu^1^, Shanli Zhu^1^, Jing Wang^1^, Yu Lin^1^, li Ma^1^, Liufang Zhu^1^, Pengyue Jiang^1^, Zhengli Li^1^, Weiqing Pan^1,2*^

**Authors’ affiliations:**

^1^ Institute for Infectious Diseases and Vaccine Development, Tongji University School of Medicine, 1239 Siping Road, Shanghai 200092, China

^2^ Department of Tropical Diseases, Second Military Medical University, 800 Xiang Yin Road, Shanghai 200433, China

**^*^ Corresponding author:**

Weiqing Pan, Ph.D., Institute for Infectious Diseases and Vaccine Development, Tongji University School of Medicine, 1239 Siping Road, Shanghai 200092, China, Work phone: 86-21-6598 5905; E-mail: wqpan0912@aliyun.com (W.P.); fax: 86-21-6598 5903.

**SUPPLEMENTARY MATERIALS AND METHODS**

**Cell Culture and Transfection**

Hepa1-6 and HepG2 cell lines were purchased from American Type Culture Collection(ATCC) and grown in Dulbecco's modified Eagle's medium(DMEM, Life Technologies, USA) supplemented with 10 %(v/v) fetal bovine serum(Invitrogen, USA), 100 U/ml penicillin, and 100 μg/ml streptomycin(Invitrogen, USA) at 37 °C in a 5 % CO2 incubator. Exponential growing cells were seeded in the culture plate overnight and transfected with 60 nM sja-miR-7-5p mimics, small interfering RNA(siRNAs) or negative control mimics(NC)(Genepharma, Shanghai, China) using Lipofectamine 3000(Invitrogen, Carlsbad, CA, USA), sja-miR-7-5p mimics: sense 5’-UGG AAG ACU GGU GAU AUG UUG UU-3’; anti-sense 5’-CAA CAU AUC ACC AGU CUU CCA UU -3’. SKP2 siRNA-786: sense 5’-GGU GAA AGC UUC AGC UCU UTT -3’; anti-sense 5’- AAG AGC UGA AGC UUU CAC CTT -3’. SKP2 siRNA-1291: sense 5’-GCC GGU GCU AUG AUA UAA UTT-3’; anti-sense 5’-AUU AUA UCA UAG CAC CGG CTT -3’. NC mimics: sense 5’-UUC UCC GAA CGU GUC ACG UTT-3’; anti-sense 5’-ACG UGA CAC GUU CGG AGA ATT-3’. The cells were incubated at the indicated time, and then subjected to further analysis as described under this section.

**In Vitro Migration Assay**

Cell migration was measured by both the Transwell migration and wound healing assay. For Transwell migration assay, cells(2×10^5^) were seeded in a 6-well plate overnight, then cells were transfected with sja-miR-7-5p mimics or NC mimics, respectively. And 24 h later, cells were digested and 2×10^4^ cells were transferred into the upper chamber in 100 μL of serum-free medium, and 500 μL compete medium with 10 %(v/v) fetal bovine serum was added into the lower chamber, three replicates per group. And 24 h later, the upper chambers were fixed in methanol for 30 minutes, followed by staining in crystal violet for 15 minutes. After stained, cells on the upper surface of the membrane were wiped out lightly by using the small cotton ball, cells on the lower surface of the membrane were photographed and counted under a light microscope in five fields. For wound healing assay, cells(3×10^5^) cells were seeded in a 6-well plate overnight and then transfected with miRNA mimics as above description. Once conﬂuent, cells were scratched in a straight line using a 200 μL sterile pipette tip. Then, suspended cells were washed off with PBS and cultured in DMEM with 1%(v/v) fetal bovine serum culture. The scratched area was photographed at 0 and 48 h, respectively. The relative area of migration formula = A/B(Where A is the area of migrated cells in experimental group after 48 h; where B is the area of migrated cells in control group after 48 h). The area of migrated cells is evaluated by the ImageJ 1.42q(ImageJ software, Way Rasband, National Institutes of Health, USA).

**RNA Preparation, Reverse Transcription, Polymerase Chain Reaction(PCR), Quantitative Real-time Reverse Transcription PCR(qRT-PCR)**

For RNA extraction, total RNA was extracted from cells, tumor samples or *Schistosoma japonicum* by using Trizol reagent(Life technologies, Carlsbad, CA, USA) according to the manufacturer's instructions, while the RNA of primary mouse hepatocytes from *Schistosoma japonicum* infected mouse was extracted by using miRNeasy Mini kit(Qiagen, Germany) according to the manufacturer's instructions. For RNA reverse transcription, miRNA were reverse transcribed into cDNA using Reverse Transcriptase M-MLV kit(Takara, Dalian, China) according to the manufacturer's instructions, and mRNA were transcribed into cDNA using PrimeScript RT Master Mix reagent kit(Takara, Dalian, China) according to the manufacturer's instructions. For quantitative real-time reverse transcription PCR(qRT-PCR), which was using SYBR green assay kit(Takara, Dalian, China) with the Applied Biosystems 7500 were performed as described previously(Zhu et al., 2016), U6 and GAPDH were used as internal control for miRNA and mRNA, respectively. The relative expression level of miRNA and mRNA was calculated by 2^-ΔΔCt^ method. All PCR assays were run in triplicate. For ordinary PCR, the amplification procedure was the same as qRT-PCR but only 32 cycles, the amplification products were performed with the 12% polyacrylamide gel electrophoresis(PAGE) at 60 volts for 3 h. The preparation of 12% polyacrylamide gel of 7.5 ml of final volume containing 3 ml 30%(w/v) polyacrylamide, 1.5 ml 5×Tris-Boric acid-EDTA(TBE), 105 μl 10%(w/v) Ammonium persulfate(APS), 5 μl N, N, N', N'-Tetramethylethylenediamine(TEMED) and 2.89 ml sterile water. After 12% PAGE, the PCR products bands were stained with the Goldview reagent(SBS genetech, Shanaghai, China) according to the manufacturer's instructions. All PCR experiments were done in triplicate. All the primers are listed in Table S1.

**Western Blot Analysis**

The Western blot analysis was performed as described previously(Hu et al., 2014). Briefly, the cell and tissue sample lysates, extracted by cell lysis buffer(Beyotime, China) and the concentration was measured by Enhanced BCA Protein Assay Kit(Beyotime, China) according to the manufacturer's instructions. About 20 μg of protein was separated by 12% SDS-PAGE and transferred to a nitrocellulose membrane, respectively. Then the membrane was blocked with 5 % bovine serum albumin(BSA) in Tris-buffered saline with Tween(TBST) for 2 h at room temperature, followed by incubated overnight with primary antibodies against SKP2(1:500 dilution, Proteintech, China), P27(1:500 dilution, Proteintech, China), MMP9(1:500 dilution, Proteintech, China), GAPDH(1:1000 dilution, Beyotime, China). After incubating with the first antibody, the membrane was washed three times with TBST, and then incubated with the relevant secondary antibodies(1:6000 dilution, Promega, USA) for 1 h at room temperature and followed by three times washes, then visualized by using the ECL reagent(GE Healthcare, UK), the protein bands were subsequently measured using the ImageQuant LAS 4000mini(GE Healthcare, USA) and grayscale analysis using the ImageJ 1.42q(ImageJ software, Way Rasband, National Institutes of Health, USA).

**Luciferase Reporter Assay**

The 3' untranslated region(UTR) wild-type(WT) and mutant(MT) of *SKP2* were amplificated from mouse genome or human genome, and then cloned into the SacⅠ/XbaⅠ site of pmirGLO Dual-Luciferase miRNA Target Expression Vector(Promega, USA), including pmirGLO-*SKP2*-WT(containing a wild type binding site in the 3' UTR of *SKP2*) and pmirGLO-*SKP2*-MT(containing a mutant type binding site), and these luciferase reporters were simultaneously transfected with sja-miR-7-5p mimics or NC mimics in Hepa1-6 or HepG2 cells, respectively. Dual-luciferase reporter assay system(Promega, USA) was used to measure the activity of the reporter gene according to the manufacturer's instructions, and the firefly luciferase activity was normalized to renilla luciferase activity.

**SUPPLEMENTARY TABLES AND FIGURES**

| **Table S1**. Sequences of primers used for qRT-PCR or ordinary PCR. | | |
| --- | --- | --- |
| **Gene** | **Name** | **Sequence(5'-3')** |
| sja-miR-7-5p | Reverse transcription stem-loop- primer RT | CTCAACTGGTGTCGTGGAGTCGGCAATTCAGTTGAGAACAACAT |
|  | Forward primer FP | ACACTCCAGCTGGGTGGAAGACTG |
|  | Common reverse primer | CTGGTGTCGTGGAGTCGGCAA |
| mmu-miR-7a-5p & hsa-miR-7-5p | Reverse transcription stem-loop- primer RT | CTCAACTGGTGTCGTGGAGTCGGCAATTCAGTTGAGACAACAAA |
|  | Forward primer FP | ACACTCCAGCTGGGTGGAAGACTA |
|  | Common reverse primer | CTGGTGTCGTGGAGTCGGCAA |
| Sja-U6 | Forward primer | CGGCGGTACATATACTAAAAT |
|  | Reverse primer | AACGCTTCACGATTTTGCGT |
|  | Reverse transcription stem-loop- primer RT | TATGGAACGCTTCACGATTTTG |
| mmu-U6 | Forward primer | GCTTCGGCAGCACATATACTAAAAT |
|  | Reverse primer & Reverse transcription stem-loop- primer RT | CGCTTCACGAATTTGCGTGTCAT |
| mmu-*Gapdh* | Forward primer | GTGTTCCTACCCCCAATGTGT |
|  | Reverse primer | GTCATACCAGGAAATGAGCTTGA |
| mmu-*Skp2* | Forward primer | GAAAGCTTCAGCTCTTTCCG |
|  | Reverse primer | AGCACCAGGAGAGATTTAGC |
| hsa-*GAPDH* | Forward primer | ACAACTTTGGTATCGTGGAAGG |
|  | Reverse primer | GCCATCACGCCACAGTTTC |
| hsa-*SKP2* | Forward primer | ATGCCCCAATCTTGTCCATCT |
|  | Reverse primer | CACCGACTGAGTGATAGGTGT |

**
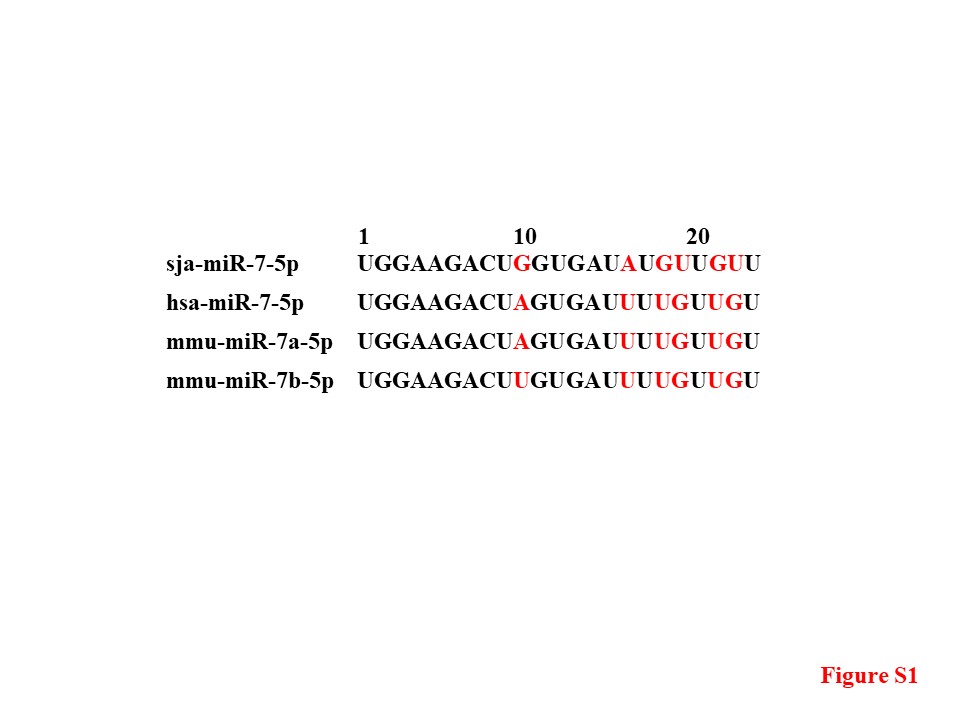
**

**Figure S1.** **Sequence alignment of miR-7-5p among *Schistosoma japonicum*, *mouse* and human.**


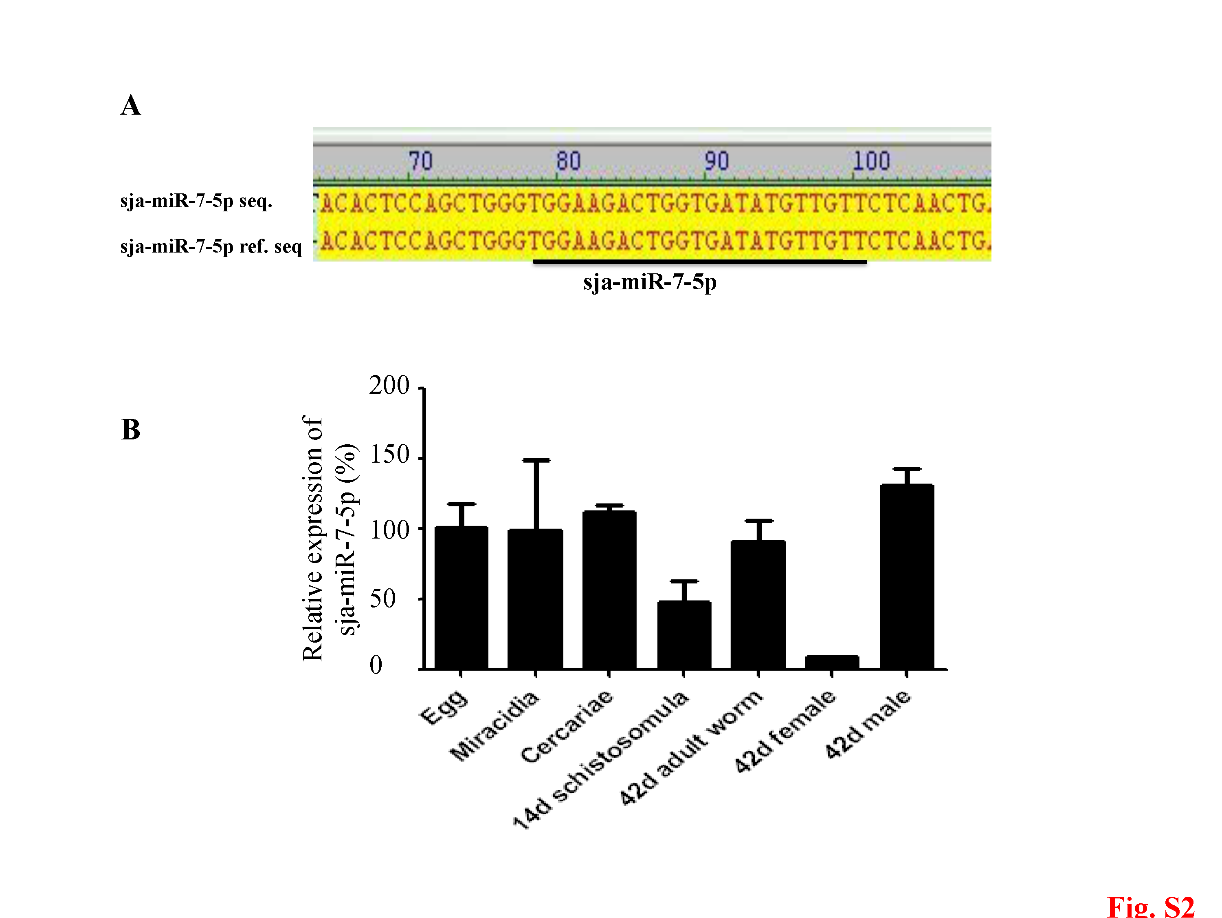


**Figure S2.** (A) The sequence alignment of the amplified mature sja-miR-7-5p in infected hepatocytes and its reference sequence. (B) Expression of sja-miR-7-5p at various developmental stages of *Schistosoma japonicum.* The miRNA expression at eggs, miracidia, cercariae, schistosomula and adult worms (male and female adult worms) were analyzed by qRT-PCR. *Schistosoma japonicum* U6 as internal control. Data were presented as Mean ± SD of three independent experiments.

**
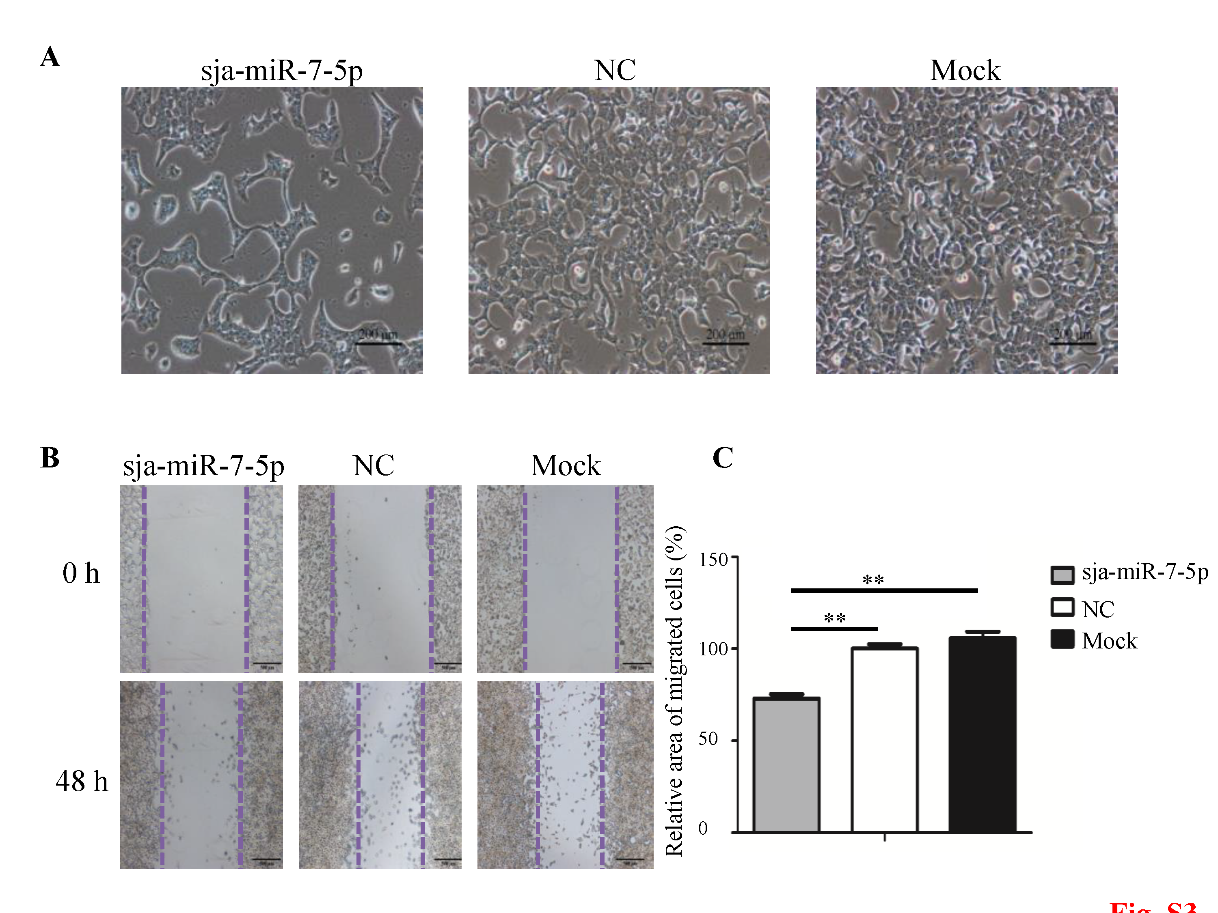
**

**Figure S3.** (A) Hepa1-6 cells were transfected with sja-miR-7-5p mimics and negative control(NC) mimics, respectively, and 48 h later, the changes in cell morphology were observed under an optical microscope. (B-C) Cell migration was evaluated using wound-healing assay. Data are presented as the mean ± SD, n = 3, **p* < 0.05, ***p* < 0.01.

**SUPPLEMENTARY REFERENCES**

Hu, C., Zhu, L.H., Luo, R., Dao, J.W., Zhao, J.P., Shi, Y.J., et al. (2014). Evaluation of protective immune response in mice by vaccination the recombinant adenovirus for expressing Schistosoma japonicum inhibitor apoptosis protein. *Parasitology Research* 113(11)**,** 4261-4269. doi: 10.1007/s00436-014-4104-5.

Zhu, S.L., Wang, S., Lin, Y., Jiang, P.Y., Cui, X.B., Wang, X.Y., et al. (2016). Release of extracellular vesicles containing small RNAs from the eggs of Schistosoma japonicum. *Parasites & Vectors* 9.
